# The emotional side of taking part in a cancer clinical trial

**DOI:** 10.1371/journal.pone.0284268

**Published:** 2023-04-24

**Authors:** Mariam Chichua, Chiara Filipponi, Davide Mazzoni, Gabriella Pravettoni

**Affiliations:** 1 Department of Oncology and Hemato-Oncology, University of Milan, Milan, Italy; 2 Applied Research Unit for Cognitive and Psychological Science, European Institute of Oncology, IRCCS, Milan, Italy; Yokohama City University, JAPAN

## Abstract

**Background:**

Taking part in a cancer clinical trial often represents a source of psychological distress and emotional activation among patients and their caregivers. Nowadays, social media platforms provide a space for these groups to freely express and share their emotional experiences.

**Aims:**

We aimed to reveal the most prevalent basic and complex emotions and sentiments in the posts of the patients and caregivers contemplating clinical trials on Reddit. Additionally, we aimed to categorize the types of users and posts.

**Methods:**

With the use of keywords referring to clinical trials, we searched for public posts on the subreddit ‘cancer’. R studio v. 4.1.2 (2021-11-01) and NRC Emotion Lexicon was used for analysis. Following the theoretical framework of Plutchik’s wheel of emotions, the analysis included: 8 basic emotions (anger, fear, anticipation, trust, surprise, sadness, joy, and disgust) and 4 types of complex emotions (primary, secondary, tertiary, and opposite dyads). We utilized the package ‘PyPlutchik’ to visualize the emotion wheels in Python 3.10.5.

**Results:**

A total of 241 posts were included in the final database. User types (129 patients, 112 caregivers) and post types (142 expressed shared experience, 77 expressed advice, and 85 conveyed both) were identified. Both positive (N = 2557, M = .68) and negative (N = 2154, M = .57) sentiments were high. The most prevalent basic emotions were: fear (N = 1702, M = .45), sadness (N = 1494, M = .40), trust (N = 1470, M = .44), and anticipation (N = 1376, M = .37). The prevalence of complex/dyadic emotions and their interpretation is further discussed.

**Conclusion:**

In this contribution, we identified and discussed prevalent emotions such as fear, sadness, optimism, hope, despair, and outrage that mirror the psychological state of users and affect the medical choices they make. The insights gained in our study contribute to the understanding of the barriers and reinforcers to participation in trials and can improve the ability of healthcare professionals to assist patients when confronted with this choice.

## Introduction

Cancer patients can see participating in a clinical trial as an opportunity with several benefits, but it also represents a source of psychological distress and emotional activation [1]. For example, patients who are offered to participate in clinical trials testing the safety or efficacy of new therapies can experience fear of the unknown [2] and the fear that they will be considered only as one experimental subject rather than a person [1]. Concerns, such as uncertainty and lack of social support, may take a severe toll on their consent to enroll and their adherence afterward [3]. This is particularly relevant if we consider that cancer elicits special needs and makes patients and their caregivers more vulnerable [4]. Indeed, experiencing cancer and the related treatment significantly impacts the patients’ lives, which may lead patients and caregivers to an overwhelming emotional activation [5].

However, even if patients highly regard clinicians for their medical knowledge and skills, they may perceive some barriers to expressing their emotional experiences to the clinicians, such as the lack of time [6] and the physicians’ lack of ‘first-hand experience’ [7, 8]. For what concerns taking part in clinical trials, sharing the negative emotional experience with their oncologist might increase patients’ fear; they may fear hurting their relationship with their doctor or receiving worse treatment in the future due to sharing their doubts [9].

Finding a safe space for expressing and sharing these emotional experiences is thus fundamental, and nowadays, social media platforms present an opportunity for patients and caregivers. Emotional needs are one of the main reasons patients turn to health-related online social platforms [10] and online health-related communities provide users with the support they cannot get from their natural social network [7]. In this regard, in a recent systematic literature review [9], social support (including emotion and network support), emotional expression, and social comparison were identified as the main categories of health-related social media use. These categories point to the psychosocial needs of patients and their caregivers and the complementary role social media plays alongside traditional healthcare facilities.

Consequently, several researchers have turned to online social media platforms, utilizing various natural language processing (NLP) techniques to analyze user posts and comments. It has been suggested that exploration of such platforms may help improve traditional psychological assessments (e.g., European Organization for Research and Treatment of Cancer [EORTC] Quality of Life Questionnaire Core 30 [QLQ-C30] and McGill Pain Questionnaire (MPQ)) that are administered to patients in medical settings and thus, address the gaps that exist when using these traditional tools [11, 12]. For example, with the help of artificial intelligence and emotion analytics algorithms, it has been possible to identify the descriptive terms of pain that have been outdated yet still used in the questionnaire and, on the contrary, the terms that are more frequently used in daily life yet not included in the assessment tool [12]. Moreover, by utilizing topic analysis of social media posts, new areas of quality of life of breast cancer patients have been described [11]. These findings demonstrate the evolving nature of language and the utility of the NLP techniques that allow for ecological qualitative online data analysis.

NLP has also been applied to explore the emotional side of online discussions. A recent sentiment analysis [13] has shown that on an online breast cancer support group, the most active users used positive language significantly more frequently than the least active users. Moreover, negative words have been significantly higher for the latter group. The difference in sentiment was evident between users with different stages of cancer as well. Similarly, the text polarity classes such as positive, neutral and negative have been the focus of another recent study that successfully predicted the sentiment of patient-authored content on e-health forums using various machine learning algorithms [14]. The importance of further research in emotion and sentiment analysis has been outlined in these works.

Focusing primarily on emotional expressions and words used online by patients and caregivers when they discuss clinical trials is important as this topic is often the source of conflicting feelings. Even though the literature on the emotion analytics of online textual material is expanding, the emotional expressions and linguistic patterns of patients and caregivers discussing clinical trials still need to be explored.

## Our aims

In this study, we aimed to conduct emotion and sentiment analysis on textual data retrieved from a public online platform serving as a cancer support group. Specifically, we were interested in the emotional experiences of those contemplating clinical trials. We approach their discussions as textual data and explore the verbal patterns representing emotional experiences expressed by users. We aimed to reveal the most prevalent basic emotions and sentiments, as well as complex emotions, in the posts of the patients and caregivers.

Second, we aimed to categorize the types of users and posts to understand better who uses this platform and what types of content are prevalent.

## Methods and materials

### Ethics statement

All posts included in the analysis are publicly available online data; therefore, ethical committee approval was not required for conducting this study. That said, we acknowledge the importance of the cautious use of public data. The use of social media data for research purposes is an emerging area, and the academic community is still developing strategies to ensure user confidentiality and consent [15, 16]. In view of these considerations, the analysis did not include names or identifiable information to ensure user anonymity.

### Data collection

The data was retrieved from the subreddit ‘cancer’ with 45.9 K subscribers. Pushshift Reddit API Documentation [17] guidelines were used to create the search string and retrieve relevant posts. The keywords included in the search were the words that may refer to clinical trials: “clinical*trial”, “new*drug”, “new*treatment”, “experimental*trial”, “experimental*drug”, “experimental*treatment”. Together with the text body of the post, metadata was downloaded (epoch, username of the poster, ID of the post, web link). Epochs were converted into human-readable dates and times.

After downloading posts based on the keywords, the databases were compared, and the duplicates were deleted. Then, the first two authors assessed the remaining posts independently for final inclusion; posts that did not focus on clinical trials were excluded.

A total of 337 public posts between July 2011 to November 2021 were identified.

### Statistical analysis

R studio v. 4.1.2 (2021-11-01) [18] was used for statistical analysis. Manual coding of each post was performed by two independent coders (C.M, C.F). This procedure consisted of labeling the type of users and posts with one of the predetermined labels. The preliminary labels (codes) were detected based on the 100 posts randomly selected by Google’s random generator. In cases of non-matching labels, the final label was chosen based on the discussion between the coders. Cohen’s kappa was calculated to determine inter-rater reliability. The values of Cohen’s kappa indicate different levels of agreement: 0 (no agreement), 0.01–0.20 (slight agreement), 0.21–0.40 (fair agreement), 0.41–0.60 (moderate agreement), 0.61–0.80 (substantial agreement), 0.81–1.00 (almost perfect agreement).

### R package ‘Syuzhet’ and the NRC lexicon

The posts from patients and caregivers were analyzed with the R package ‘Syuzhet’ [19]; this package represents a sentiment extraction tool developed in the Stanford NLP group. It allows users to work on the sentiment data in their text files. Emotions and sentiments analysis was performed using the function ‘get_nrc_sentiment’, which considers eight basic emotions and two sentiments. This function uses NRC Emotion Lexicon [20, 21]. The lexicon is a list of words, each associated with corresponding emotions (anger, fear, anticipation, trust, surprise, sadness, joy, and disgust) and sentiments (positive and negative); this classification is in line with the work of Plutchik and his wheel of emotions [22]. The NRC lexicon has been increasingly applied in recent years for quantifying affect in online textual data [23–25].

With the use of ‘get_nrc_sentiment’ function, words of the data can be matched with identical words in the NRC lexicon and described by the emotions and sentiments they represent. Once the ‘get_nrc_sentiment’ function is applied to the text, a table is returned to the user, and later the returned table can be accessed and analyzed as any other data frame. This data frame consists of the split text arranged in rows, while the columns contain the corresponding sentiment and emotion association. The association’s presence can be determined by the number ‘0’ or ‘1’, where ‘0’ indicates that the word is not associated with the emotion or sentiment, whereas ‘1’ confirms the association. A word can be related to more than one emotion and may have a positive, negative, or polarity orientation. For example, words that embody anger, fear, disgust, and sadness are mostly associated with negative sentiment.

On the contrary, words expressing anticipation, joy, and trust are primarily associated with positive sentiment. Furthermore, surprise words can be either positive or negative, representing both polarities. Table 1 illustrates 10 example words from our dataset and how they are codified into the eight basic emotions in the lexicon.

**Table 1:** Example of 10 words extracted from the NRC Lexicon.

*Hospital*	positive:0	negative:0	anger:0	anticipation:0	disgust:0	fear:1	joy:0	sadness:1	surprise:0	trust:1
*Curable*	positive:1	negative:0	anger:0	anticipation:0	disgust:0	fear:0	joy:0	sadness:0	surprise:0	trust:1
*Time*	positive:0	negative:0	anger:0	anticipation:1	disgust:0	fear:0	joy:0	sadness:0	surprise:0	trust:0
*Hope*	positive:1	negative:0	anger:0	anticipation:1	disgust:0	fear:0	joy:1	sadness:0	surprise:1	trust:1
*Pain*	positive:0	negative:1	anger:0	anticipation:0	disgust:0	fear:1	joy:0	sadness:1	surprise:0	trust:0
*Death*	positive:0	negative:1	anger:1	anticipation:1	disgust:1	fear:1	joy:0	sadness:1	surprise:1	trust:0
*Risk*	positive:0	negative:1	anger:0	anticipation:1	disgust:0	fear:1	joy:0	sadness:0	surprise:0	trust:0
*Promise*	positive:1	negative:0	anger:0	anticipation:0	disgust:0	fear:0	joy:1	sadness:0	surprise:0	trust:1
*Failure*	positive:0	negative:1	anger:0	anticipation:0	disgust:1	fear:1	joy:0	sadness:1	surprise:0	trust:0
*Cancer*	positive:0	negative:1	anger:1	anticipation:0	disgust:1	fear:1	joy:0	sadness:1	surprise:0	trust:0

## Plutchik’s wheel of emotions

According to Plutchik [22], each of the basic emotions (out of 8) forms a complex emotion (dyad) when it is combined with one of the other 7 basic emotions. Plutchik visualized basic emotions on a wheel, allowing us to understand better how basic emotions are related to one another based on their spatial placement. Considering how far the two co-occurring emotions are, they form primary, secondary, and tertiary dyads or opposites.

Following this background, we extracted the frequencies of basic emotions to calculate the co-occurrence between emotions for creating the primary, secondary, tertiary, and opposite emotions’ dyads. Specifically, the co-occurrence in a sentence was represented by the presence of each specific emotion associated with another one. For example, joy and sadness are opposites, forming a complex emotion, ‘bitter sweetness’. Therefore, if in a sentence, the frequency of sadness is 2 and the frequency of joy is 3, the frequency of bitter sweetness was set to 2. Thus, the sum of each co-occurrence in a sentence was calculated as a representation of the frequency of a specific dyad.

To visualize the wheel, Python 3.10.5. was used [26]. We utilized the package ‘PyPlutchik’, a novel Python implementation designed to visualize Plutchik’s wheel of emotions [27]. For each wheel (basic emotions, primary dyads, secondary dyads, tertiary dyads, opposites) the emotion of the highest frequency was selected and set to 1. The scores of the rest of the emotions were calculated based on this ratio. As a result, emotions on the wheel range from 0 to 1, where 1 is the most frequent emotion.

## Results

A total of 337 public posts between July 2011 to November 2021 were identified. Inter-rater reliability manual coding rated a perfect agreement (from .98 to .99) for both broad categories (see Table 2).

241 (129 from patients, 112 from caregivers) out of 337 comments were included in the final database since our aim was focused on patients and caregivers. Therefore, 96 comments were excluded from the analysis since the user type was unknown (N = 89) or referred to healthcare professionals (N = 7). See Table 2.

**Table 2:** List of broad categories, related code, and inter-rater reliability analysis.

Broad category	Code	Frequency (%)	Inter-rater reliability (N = 337)
**Type of users**	Patient	129(38.3)	
	Caregiver	112(33.2)	K = .99***
	HC^a^	7(2.1)	
	Unknown	89(26.4)	
**Type of comments**	Advice	77(22.7)	
	Experience	142(41.9)	K = .98***
	Both^b^	85(25.1)	
	Unknown	35(10.3)	

^a^healthcare professionals

^b^advice and experience.

### Sentiments and emotions analysis

**Fig 1:**
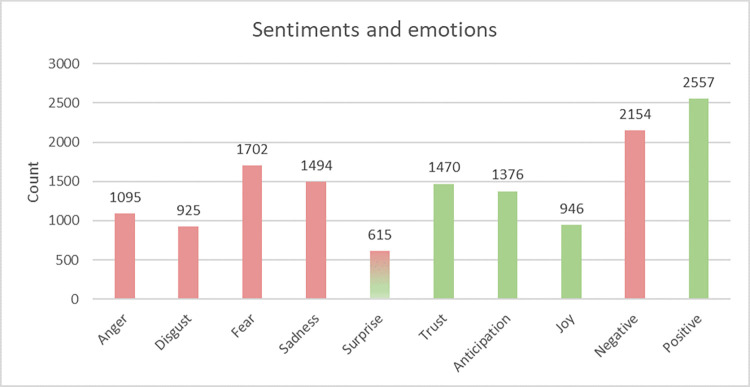
Frequencies of patients’ and caregivers’ sentiments and emotions textually expressed when discussing clinical trials. The negative polarity of emotions and sentiments is indicated in red, and the positive ones–in green. Both red and green were used for emotions that may be associated with both negative or positive emotions and sentiments. The count represents the total frequency of association between the word and one or more emotions and/or one of two polarities (negative and positive) present in the data body text.

Fig 1 shows the most prevalent emotions and sentiments in the posts of patients and caregivers when sharing advice, experience, or both regarding clinical trials. The total length of the words was 62061.

Both *positive* (N = 2557, M = .68) and *negative* (N = 2154, M = .57) sentiments were high, with a slight prevalence of target words associated with the positive sentiment. Regarding the positive category, *trust* (N = 1470, M = .44) and *anticipation* (N = 1376, M = .37) were the two most prevalent emotions, followed by *joy* (N = 946, M = .25). Regarding the negative category, *fear* (N = 1702, M = .45) and *sadness* (N = 1494, M = .40) were found to be the highest, followed by *anger* (N = 1095, M = .29) and *disgust* (N = 925, M = .25).

On the contrary, users’ lowest affect expressed in the posts was *surprise* (N = 615, M = .16).

Tables 3 and 4 show examples of extracts from posts of users with the sentiments and emotions associated with each target word. Specifically, Table 3 presents an extract from a patient’s post sharing their experience. The rows represent sentences from their post, and the columns are created for emotions and sentiments. The numbers show how many times a specific emotion or sentiment was expressed in a given sentence. In Table 4, the columns represent emotions and sentiments. In contrast, the rows are examples (extracts) of different posts, post ID/user type, and the frequency of the specific emotion or sentiment (based on the column) detected in the example extract.

**Table 3:** Twelve extracted sentences from post ID 59 (user type–type of comment: Patient–experience) along with the sentiments and emotions frequencies for each sentence.

Sentences	Anger	Anticipation	Disgust	Fear	Joy	Sadness	Surprise	Trust	Negative	Positive
[1] *"Luckily after that*, *experimental treatments seemed to have tempered my cancer*, *since then it was small recurrences*.*”*	1	0	1	1	0	1	0	0	2	1
*[2] "Granted, this stole my childhood, adolescence, and early 20’s, but in my mind, there is an abundance of hope, just as there is of hopelessness.”*	1	2	2	1	3	1	1	2	2	4
[3] *"I would strongly urge you to acknowledge the existence of both*.*”*	0	0	0	0	0	0	0	0	0	2
[4] *"Sure*, *treatment or surgery could fail*, *be inaccessible*, *and you could die*.*"*	0	0	0	2	0	2	0	0	2	0
[5] *"But so long as you keep fighting*, *giving it your all*, *you give yourself a chance at surviving*.*”*	1	1	0	0	0	0	1	0	1	1
[6] *"It is a truly dark and miserable situation to be in*, *and loss*, *as well as the threat of loss*, *is debilitating*.*”*	3	0	1	2	0	3	0	0	3	0
[7] *"I was only a kid*, *and lived a brutally awful childhood prior*, *so I didn’t have much to lose*.*”*	2	0	2	2	1	2	1	0	2	1
[8] *"Recurrences were more taxing when I had built up a life of my own*, *and I can only imagine the stress that it is bringing you; to threaten the sanctity of things so sacred to you as your daughter*.*”*	1	1	0	1	1	0	0	0	2	1
[9] *"As to what’s to come*?*"*	0	0	0	0	0	0	0	0	0	0
[10] *"Another long brutal fight*, *possibly more hopeless than the last*, *but the important thing is that miracles do happen*, *my survival chance was in the single digits back when any doctor was willing to assign a number to it*, *and they have long since forsaken trying to numerically explain that*, *as by all logic and reason*, *I should not be alive*.*"*	3	3	0	3	1	2	1	4	4	5
[11] *"Just a question of if you can keep things from going terminal and maintain your will to continue fighting as hard as you may need to get there*.*”*	1	1	0	1	0	1	0	1	2	2
[12] *"I would so highly recommend building and reinforcing a support network as much as you can and figure out what their limits are so that you don’t over-tax and exhaust any parts of it*.*”*	0	1	0	0	0	1	0	1	2	2

*Note*. 0 represents that a target word is not associated at all with one or more emotions and/or one of the two polarities (negative or positive), and 1 represents that there is an association between the target word and one or more emotions and/or one of the two polarities (negative or positive).

**Table 4:** Extracts from posts representing the emotions.

Emotion	Trust	Fear	Anticipation	Sadness	Joy	Anger	Disgust	Surprise
**Examples**	*“…Not all* ***hope*** *is desperate*, *I’m optimistic about new treatments for TNBC not out of irrational desperation*, *but because science has made amazing advances in the field*, *and I want to at least have the chance for* ***medical*** *science to* ***help*** *my wife beyond what standard chemotherapy can do*. *Not a* ***miracle*** *cure—just real science that gives me* ***hope***.*”*	*"This* ***disease*** *is* ***terrible***..* *.* *.*After many scans my mother was told her* ***case*** *was special and very hard to battle*..* *.* *. *As of recently*, *my mother has been on an experimental chemo pill that has helped extend her life but not resolved the* ***tumor*** *growth*..* *.* *.*She’s currently going blind*, *and her eyes are basically being pushed out of her skull by the* ***tumors****—it’s* ***horrific*** *to watch them grow so fast and there’s nothing we can do*..* *.* *. *Get second opinions and don’t be* ***afraid*** *to tell a doctor when they are being heartless*. *Everywhere we go* ***medical*** *staff* ***treat*** *her as a walking dead person*.*"*	*"… he is considered an "ideal" candidate because other than being a renal tumor factory*, *he’s in spitting* ***good*** *shape- no other medications*, *no recent chemo- he’s a blank slate for them to* ***experiment*** *on*. *… ultimately*, *every* ***patient*** *(and family) has to make his or her own decision*. *i’m* ***glad*** *you didn’t let your dad get pressured into a decision he wasn’t* ***ready*** *to make; i* ***wish*** *you the best of outcomes on this part of your* ***journey***.*"*	*“…The way I see it*, *there’s not much of anything that they can do to me that will be* ***worse*** *than* ***dying*** *of asshole* ***cancer*** *(I was real close*.* *.* *.*I know what the end is like)*, *so I’m up for pretty much anything*, *testing-wise*. *If* ***killing*** *me with some new experimental drug prevents them from having to* ***kill*** *someone else with it to find out it’s* ***lethal*** *to humans*, *then I’m down*.*”*	*"*.* *.* *. *There is so much research and experimental treatment going on out there these days*, *you have to jump at every chance you’re offered for her*. *I’m truly* ***blessed*** *for the chance I had with car-t cells*. *I’m not sure what type of cancer your wife has*, *but it has been a* ***godsend*** *to my refractory leukemia*. ***Thank*** *you for the* ***happy*** *thoughts*! *… I am living proof that they work and as I’m sad I’m not dead quite yet*, *it has shown me to be very* ***thankful*** *for the* ***progress*** *I’ve been able to make*.*"*	*"…Why did the doctor who worked on my nephew who had* ***cancer*** *try to put him in an experimental treatment that had a 71% chance of* ***death***? *… With the parents no longer in the way*, *and my nephew in a foster home for 8 months*, *the doctor who had full authority to now try the experimental* ***cancer*** *treatment ended causing my nephew to have not 1 but 2 seizures*, *effectively* ***destroying*** *his speech and causing him to walk funny*. *… The* ***greed*** *of man for centuries has caused pain and* ***death*** *to billions of people*.*"*	*“I* ***finally*** *had to just tell my doctors that I wasn’t interested in that* ***shit*** *anymore and that either they were going to put me back on the* ***failed*** *drugs to try to stabilize*, *or I was going to find a new doctor that would*.*”*	“*At this point*, *you and hubby are still in* ***shock*** *about his diagnosis*. *… Lung cancer is a very common cancer which means there is lots of research going on and oncologists see it all the time so its no* ***mystery*** *to them how to* ***treat*** *it…*. *Doctors may* ***quote*** *some length of time based on data for particular cancer and their experience*. *It’s just a best* ***guess***.*”*
**Post ID / use type**	*47 / caregiver*	*227 / caregiver*	*65 / caregiver*	*50 / patient*	*60 / patient*	*61 / caregiver*	*49 / patient*	*72 / patient*
**Frequency**	*5*	*8*	*7*	*6*	*6*	*6*	*3*	*5*

In italics-bold is the target word (i.e., the word for which emotion associations are provided).

### Plutchik’s wheel of emotions

**Fig 2:**
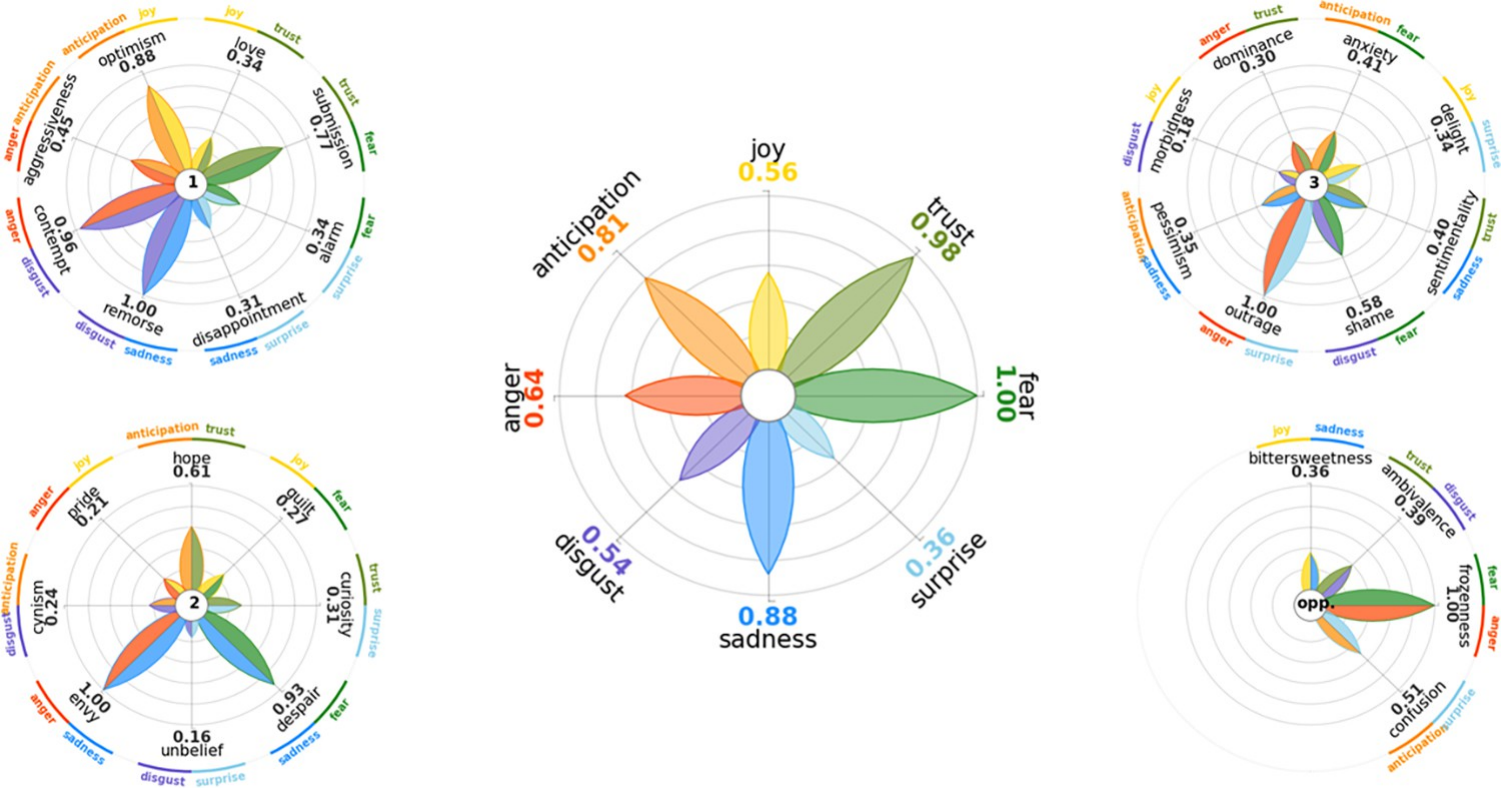
Plutchik’s wheels of emotions. The central wheel shows basic emotions, the wheel in the upper left corner shows primary dyads, the wheel in the lower left corner shows secondary dyads, the wheel in the upper right corner shows tertiary dyads, and the wheel in the lower right corner shows opposites.

The frequency of co-occurrence between pairs of emotions is reported in Table 5. The three most frequent primary dyads were remorse, contempt, and optimism, followed by submission, aggressiveness, love, alarm, and disappointment. Regarding the secondary dyads, the two most frequent were envy and despair, followed by hope, curiosity, guilt, cynism, pride, and unbelief. The most frequent tertiary dyad was outrage, followed by shame, anxiety, sentimentality, pessimism, delight, dominance, and morbidness. Finally, regarding the opposite dyads, we found frozenness to be the most frequent pair of emotions, followed by confusion, ambivalence, and bittersweetness. Refer to Fig 2 to view the wheel of emotions created by PyPlutchik Python package [27] based on our data.

**Table 5:** The co-occurrence of emotions.

Dyads		Emotions association	Co-occurrence frequency	Ratio
**Primary**	**Remorse**	Disgust+Sadness	813	1
	**Contempt**	Anger+Disgust	778	0.96
	**Optimism**	Anticipation+Joy	719	0.88
	**Submission**	Trust+Fear	626	0.77
	**Aggressiveness**	Anger+Anticipation	370	0.45
	**Love**	Joy+Trust	279	0.34
	**Alarm**	Fear+Surprise	276	0.34
	**Disappointment**	Sadness+Surprise	254	0.31
**Secondary**	**Envy**	Anger+Sadness	1322	1
	**Despair**	Sadness+Fear	1225	0.93
	**Hope**	Anticipation+Trust	802	0.61
	**Curiosity**	Trust+Surprise	408	0.31
	**Guilt**	Joy+Fear	356	0.27
	**Cynism**	Disgust+Anticipation	325	0.24
	**Pride**	Anger+Joy	275	0.21
	**Unbelief**	Surprise+Disgust	207	0.16
**Tertiary**	**Outrage**	Anger+Surprise	1375	1
	**Shame**	Disgust+Fear	796	0.58
	**Anxiety**	Anticipation+Fear	568	0.41
	**Sentimentality**	Sadness+Trust	553	0.40
	**Pessimism**	Anticipation+Sadness	485	0.35
	**Delight**	Joy+Surprise	462	0.34
	**Dominance**	Anger+Trust	417	0.30
	**Morbideness**	Disgust+Joy	244	0.18
**Opposite**	**Frozenness**	Fear+Anger	944	1
	**Confusion**	Anticipation+Surprise	482	0.51
	**Ambivalence**	Trust+Disgust	369	0.39
	**Bittersweetness**	Joy+Sadness	344	0.36

## Discussion

Online platforms provide a space for users to unload their tension and emotional discharge without taking responsibility for the effects this may have on others. In regard to emotional needs, social media platforms may satisfy the need for both emotional support (the communication between two or more users in which they mutually share and discuss their affective needs) and emotional expression (the freedom with which patients can disclose their experiences, regardless of if someone responds to them, without the concern that the shared experience will impact others around them) [28–30]. Addressing these needs is especially important, considering that some users, such as cancer patients and their caregivers, tackle emotionally charged challenges daily. Previous studies have explored posts on online cancer communities with methods such as topic analysis [11] and sentiment analysis [13, 14]. Our study differs from the previous works in two main aspects: (1) We narrowed our focus to the experiences of patients and caregivers discussing clinical trials. Due to their experimental, innovative, and at times risky nature, clinical trials may evoke particularly challenging emotions in users. Therefore, non-specific discussions in cancer communities may not fully reflect this topic’s nuance. (2) Given the complexity of these emotions, our analysis goes beyond the polarity of sentiments. In our analysis, we included the 8 basic emotions, following the theoretical framework of Plutchik and dyadic emotions. This way, we bridge the natural language processing algorithms with a psychological theoretical framework of emotions.

Overall, this study points out that users shared two main types of content in this online support group: personal experience and advice. Additionally, we found an almost equal distribution of patients and caregivers among posts with an identifiable user type. This, in our view, stresses that patients neither experience the clinical trial-related distress alone nor make decisions in isolation; in most cases, the caregivers are part of all the steps. Furthermore, caregivers may have an even higher burden than patients in trials [31], and elevated levels of commitment may impact the caregiver’s ability to provide care for the patient [32]. Therefore, we consider focusing on patients’ and caregivers’ emotions essential.

This study identified the most prevalent basic and complex emotions underlying users’ posts on the subreddit ‘cancer’. As represented in Plutchik’s wheel (Fig 2), the most prevalent basic emotions expressed in texts were fear, trust, and sadness. These emotions may correspond with the innovative nature of oncological clinical trials. Still, in the experimental phase, the drug’s side effects are not always predictable or obvious [33]. Therefore, trials represent the unknown and may elicit fear, concerns about trust towards the new drug or the physician responsible for the trial, and sadness resulting from the overall confusion. Previous works with patients in the trial or who are considering joining one inform us that patients often fear the side effects of clinical trials and the unknown surrounding these studies [34]. The fear that has been previously reported is about being treated as a “guinea pig” (being used for the trial and the career of the principal scientist, with no personal benefit) [35]. This fear was mirrored in our database as well; one of the caregivers shared:

*“The clinical trial my brother is in has no efficacy because there were no trials. Basically, he’s a well-supported lab rat…”*

This post not only represents the users’ concern but may also represent fear and trust issues towards the healthcare system and the sadness evoked by the confronted reality. Similar emotions may be elicited due to other concerns surrounding clinical trial participation. Among the barriers that hinder patients from participating are treatment-related concerns, fear of side effects, financial concerns, dislike for the experimental setting, and random assignment of patients to experimental and placebo groups [36].

Considering the complex emotions created by the dyads (Fig 2), we saw that among the most prevalent ones were (here we list those with a ratio above 0.8): remorse (composed of disgust and sadness); contempt (composed of anger and disgust); optimism (composed of anticipation and joy); envy (composed of anger and sadness); despair (composed of sadness and fear); outrage (composed of anger and surprise); and frozenness (composed of fear and anger). Most of these complex emotions (apart from optimism) are considered negative. This again emphasizes the struggles that patients and caregivers face and the emotional turmoil they go through. However, while studies focus on some psychological challenges faced by patients going through trials (e.g., lowered quality of life [37]), there are no studies investigating complex dyadic emotions, apart from optimism, in clinical trials. Some studies focus on optimism and clinical trials, specifically, unrealistic optimism about the outcomes of participation in a trial [38, 39].

We believe that the prevalence of dyadic emotions mirrors the complexity of clinical trial-related experiences. Clinical trials may have different meanings given to them by different people. While some relate it to being a “lab rat” (as in the post of a caregiver above), others may give it a completely different sense, such as the one provided by another user in our data:

*“…I’ve had similar thoughts, and I’ve concluded that if I relapsed again, I would do a clinical trial still, not for myself, but because it would provide valuable data for future patients whether or not it works for me. Do with that what you will.”*

Given that trials can be associated with altruistic narratives such as the one above (i.e., helping future patients), declining to participate in such research can elicit feelings of regret and guilt and the perception of personal moral failure [40, 41]. Moreover, this decision’s emotional burden may become even more complex due to the patient’s relationship with their oncologist. Many patients have a long relationship with their oncologist, and declining participation may elicit fear of harming this rapport or even cause worse treatment in the future [9]; such complex experiences may result in dyadic emotions (e.g., despair and frozenness).

Investigating complex emotions is crucial as it may reveal nuances that cannot be accessed by solely focusing on basic emotions. For example, an interesting observation we made in our results is about anger. Even though anger was not among the most frequently expressed basic emotions (being the fifth among eight basic emotions, Fig 1), within the most frequent dyadic couples (creating a complex emotion), anger is a recurring primary emotion. More specifically, anger is in the composition of the following: contempt, envy, outrage, and frozenness. This finding points to the fact that users experience anger about clinical trials in complex ways, often coupling it with other emotions. This may be due to the conceptual complexity of clinical trials; the diversity of the aspects that users consider when discussing them; suppression of direct expression of anger, and limiting it to be one of the experiences, among many others, etc.

We want to stress the importance of focusing on the emotional experiences of patients and their caregivers for further research. It is relevant not just for the overall well-being of these groups but also for gaining a deeper understanding of their behavioral patterns when deciding on their health. For example, it has been demonstrated among oncological patients that fear, anxiety, and worry interfere with medical decisions and behavior [42]. Understanding the mechanisms behind medical choices has critical implications on an individual patient level, as it can improve the ability of healthcare professionals to assist patients when confronted with a medical decision. Furthermore, considering the role of emotions in medical decision-making can affect the development of the field on a large scale. This is especially true for cancer clinical trials as the main gateway to developing new anti-cancer treatments. The most prevalent barriers to conducting a clinical trial are patients’ concerns about disease progression, experienced side effects, and dropout due to personal issues [43]. Therefore, it is crucial to consider the emotional experiences of patients and their caregivers, as they could interfere with their willingness to participate in or continue the clinical trial.

This study has some limitations. First, we did not compare patients’ and caregivers’ emotional experiences. Further research should consider this limit and assess emotions separately to explore their different perspectives.

Second, we needed access to the demographics and personal characteristics of Reddit users; therefore, differences based on these variables could not be considered. Moreover, within the methodological approach of this study, emotions cannot be linked to specific experiences about trials; in this respect, we can only speculate about possible explanations. This study aimed to reveal the prevalent emotions and sentiments rather than investigate their link to specific experiences. Nevertheless, this may present an interesting direction for future research.

## Conclusions

In summary, although the barriers to participation in trials have been primarily investigated in the literature [3, 43], the nature of emotions standing behind patients’ and caregivers’ concerns has not been thoroughly explored. With this study, we address the question of the most prevalent basic and complex emotions among patients and caregivers discussing cancer clinical trials, emphasizing the importance and relevance of their diverse outlooks.

Finally, we would like to conclude this paper with a post from one of the users. The perspective portrayed in the post encapsulates the complexity of experiences in clinical trials and the complexity of emotions accompanying them:

*“…The thing with trials is that there’s no guarantee that it’s going to work. It may extend your life, it may even shorten your life, or you may end up in the placebo group where you’re not actually getting the medicine on trial. Is it worth it? That’s really down to an individual’s feelings about it. Some people will continue to grasp at straws no matter how dire their chances may be. Some people would rather spend their remaining time with their families. Others want to contribute to the advancement of treatments so others after them may have better chances of survival.”*
